# Role of Multisector Partnerships in Controlling Emerging Zoonotic Diseases

**DOI:** 10.3201/eid1112.051322

**Published:** 2005-12

**Authors:** Nina Marano, Paul Arguin, Marguerite Pappaioanou, Lonnie King

**Affiliations:** * Centers for Disease Control and Prevention, Atlanta, Georgia, USA; † University of Minnesota, Minneapolis, Minnesota, USA; ‡ Michigan State University, East Lansing, Michigan, USA

**Keywords:** emerging zoonoses, infectious diseases, multisectorial, partnerships, surveillance, wildlife, disease emergence, animal health, human health, introduction

**Figure 1:**
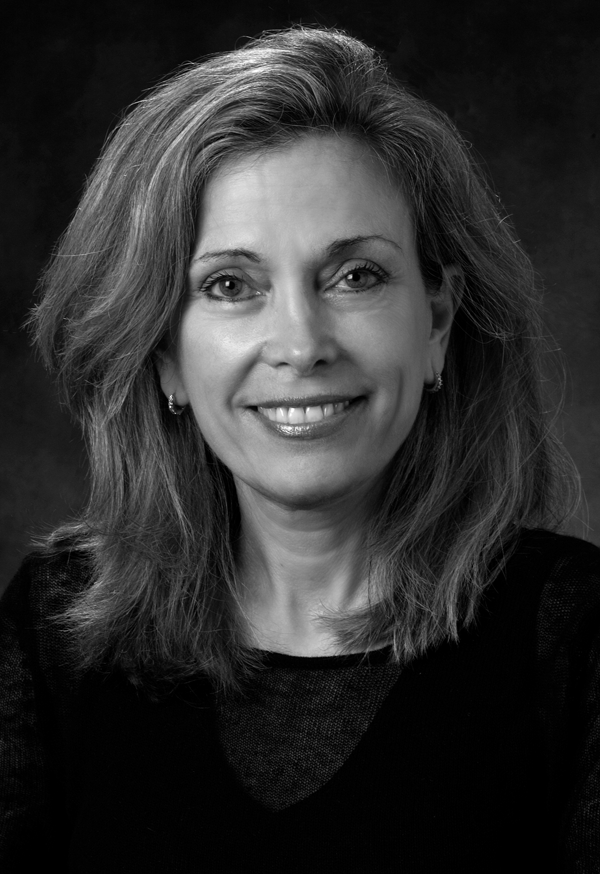
Photo of Nina Marano. Dr Marano is the associate director of veterinary public health in the Division of Bacterial and Mycotic Diseases, CDC. She is responsible for promoting multisector partnerships to enhance detection, prevention, management, and control of emerging zoonotic diseases.

**Figure 2:**
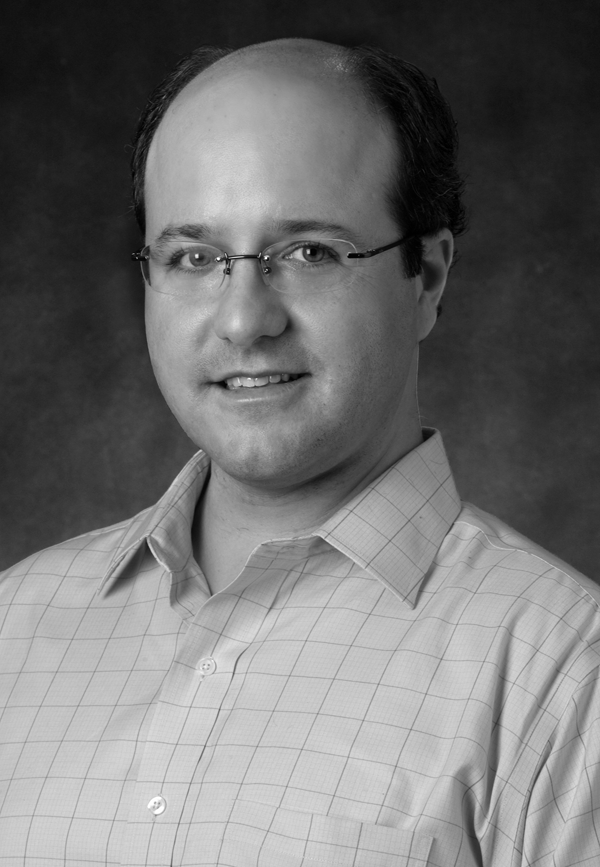
Photo of Paul Arguin. Dr Arguin is the acting chief of the Geographic Medicine and Health Promotion Branch and the zoonoses team leader in the Division of Global Migration and Quarantine, CDC. The team's mission is to prevent the introduction of zoonotic diseases into the country through imported animals and animal products.

**Figure 3:**
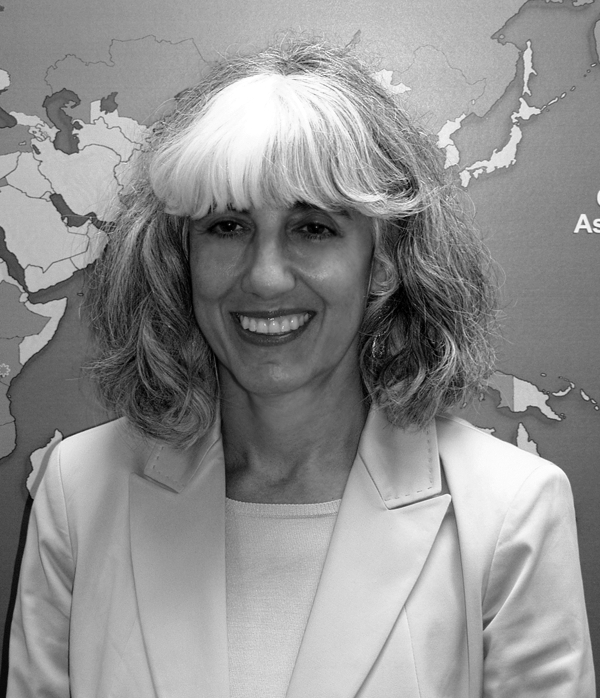
Photo of Marguerite Pappaioanou. Dr Pappaioanou is professor of infectious disease epidemiology in the School of Public Health with a joint appointment in the College of Veterinary Medicine at the University of Minnesota. Her areas of interest are in emerging zoonotic infectious diseases, with a special interest in influenza viruses and in collaborative efforts that bridge public health and domestic animal and wildlife health sectors that address emerging zoonotic infectious diseases.

This issue marks the second time that an issue of Emerging Infectious Diseases has been devoted to zoonotic diseases; the first zoonoses issue was published 1 year ago, in December 2004. The publication of this second theme issue attests to the frequency, visibility, and attention that these diseases are receiving. A year ago, we (Figures 1, 2, 3) commented on several prevailing factors worldwide that facilitate the emergence of zoonotic infectious diseases, among them a growing human population, increased interaction between species, global climate changes, and rapid movement of people and animals (*1*). These factors continue to exert their influence, and we continue to see a plethora of emerging zoonotic infectious diseases.

In their book Beasts of the Earth: Animals, Humans, and Disease, Torrey and Yolken point out that domestic and international public health and animal health agencies have a long history of poor coordination and little effort to bridge the gulf between these 2 professional worlds (*2*). The authors suggest that we must learn to cooperate if we are to effectively combat emerging microbial threats. In the past year, improved cooperation has been evident. We have observed early detection and response to several important zoonotic diseases threatening the public's health. These responses were made possible by several strategic partnerships across human and animal health sectors—partnerships that have been long in the making.

As this issue goes to press, the year has been bracketed by several major natural disasters in 2 hemispheres—the tsunami in Southeast Asia, hurricanes in North America, and the earthquake in Pakistan and India. These events underscore the fragility of our society and the importance of working in partnerships to effectively protect and promote the health of all persons in challenging times. In the United States, understanding the potential threat for zoonotic disease outbreaks in natural disaster settings, local and state agencies and the Centers for Disease Control and Prevention (CDC) have worked in partnership with nongovernmental and other federal agencies to augment surveillance systems to allow for early detection and response to potential rodent- and insect-borne infectious disease threats (*3*).

In between these events, the world detected and responded to a range of emerging microbial threats from all corners of the animal kingdom, including wildlife, captive wildlife in zoos, domestic poultry and livestock, and pet animals (*4*). Recurring reports have shown that H5N1 avian influenza in Southeast Asia is moving into eastern Europe, and scientists are concerned that this virus could rapidly move across geographic regions through poultry, animal husbandry, and wild bird migration (*5**,**6*). Outbreaks of *Escherichia coli* have been detected in petting zoos (*7*). Lymphocytic choriomeningitis and West Nile virus have been transmitted through organ transplantation, and outbreaks of *Salmonella* spp. have been traced back to pet rodents (*8**–**10*). The world also witnessed the remarkable survival of a young woman with rabies in Wisconsin (*11*).

The articles in this special themed issue reflect emergence and reemergence of a wide array of known zoonotic pathogens, including lyssavirus, hantavirus, Rift Valley fever, methicillin-resistant *Staphylococcus aureus*, *Echinococcus* spp., norovirus, Nipah virus, and *Bartonella* spp., as well as pathogens for which the potential for spread to humans is yet unknown, such as canine influenza virus and phocine distemper virus (*12**–**14*).

How should we respond to these emerging disease challenges? This year has brought about renewed, and at times unprecedented, collaborations and partnerships to confront these health challenges. Wildlife, animal agriculture, and public health agencies worked together, often for the first time. They developed surveillance plans for monitoring wild birds for highly pathogenic avian influenza (HPAI), provided guidance for safely handling wild birds during these monitoring efforts, and created a comprehensive plan to combat avian flu in Southeast Asia. Such partnerships also facilitated collection of human and wild bird specimens for HPAI H5N1 surveillance in Southeast Asia, use of a survey instrument to evaluate state animal health–human health communication and coordination, and collaborations with industry for recommendations for safely handling pet rodents (15, N. Marano (Figure 1), unpub. data).

However, we need to respond further by calling for more multidisciplinary, integrated research that identifies the causes and factors leading to the emergence of zoonotic diseases and explores how to effectively prevent and control them (*16*). Avian influenza, in particular, has shown the importance of this research, as the results are vital to the health of both human and animal populations.

In 2006 we look forward to strengthening and nurturing essential collaborations between organization to improve human and animal health. One step will be the International Symposium on Emerging Zoonoses, organized by the World Animal Health Organization and CDC, to be held in Atlanta in March 2006.

This past year we have begun to come together. Let us do everything we can to continue in this direction, and the reward will be success in protecting and promoting human and animal health through effectively confronting zoonotic infectious diseases. This theme issue is an important component in this process.
